# Albumin-to-protein ratio in spot urine samples for analysis of proteinuria selectivity in chronic kidney disease

**DOI:** 10.1590/2175-8239-JBN-2022-0079en

**Published:** 2022-10-03

**Authors:** Miguel Augusto Martins Pereira, Roger Freitas Ramirez Jordan, Jorge Paulo Strogoff de Matos, José Carlos Carraro-Eduardo

**Affiliations:** 1 Universidade Federal Fluminense, Escola de Medicina, Niterói, RJ, Brazil; 2 Universidade Federal Fluminense, Escola de Medicina, Departamento de Nefrologia, Niterói, RJ, Brazil

**Keywords:** Urine, Proteinuria, Electrophoresis, Albuminuria, Renal Insufficiency, Chronic, Insuficiência Renal, Crônica, Prevenção de Doenças, Conhecimento, Características da População.

## Abstract

**Introduction:**

The albumin-to-creatinine ratio and total protein-to-creatinine ratio in spot urine samples have already been validated as surrogates for 24-hour albuminuria and proteinuria measurements. Thus, we hypothesized that the type of proteinuria, detected by the electrophoretic pattern of 24-hour urine, could be predicted by the simple proportion of albumin in the total urine protein content, using the albumin-to-protein ratio (APR). Our study sought to validate the use of APR as a cheaper substitute for urinary protein electrophoresis (UPE).

**Methods:**

Using different mathematical models, we compared, the albumin fraction in 24-hour urine samples by electrophoresis and the APR ratio in spot samples from 42 outpatients with chronic kidney disease (CKD).

**Results:**

A strong log-order correlation r = 0.84 (0.75–0.92; 95% CI, p = 0.001) was observed between APR and the albumin fraction in the UPE.

**Conclusion:**

The APR can substitute electrophoresis in CKD outpatients.

## Resumo

**Introdução:**

A utilização da razão albumina/creatinina e da razão proteína total/creatinina em amostras isoladas de urina já foram validadas como substitutos para a albuminúria e proteinúria em 24 horas. Assim, nossa hipótese é que o tipo de proteinúria, dado pelo padrão eletroforético da urina de 24 horas, poderia ser previsto pela simples proporção de albumina no conteúdo total de proteínas na urina, utilizando a razão albumina/proteína (RAP). O presente estudo procurou validar o uso da RAP como um substituto mais prático e de menor custo da eletroforese de proteínas urinárias (EPU).

**Métodos:**

Foram utilizados diferentes modelos matemáticos a fim de comparar a fração de albumina pela eletroforese em amostras de urina de 24 horas e a RAP em amostras isoladas em 42 pacientes ambulatoriais com doença renal crônica.

**Resultados:**

Foi observada uma forte correlação logarítmica r = 0,84 (0,75–0,92; 95% CI, p = 0,001) entre a RAP e a fração de albumina pela EPU.

**Conclusão:**

A RAP pode substituir a eletroforese urinária em pacientes renais crônicos ambulatoriais.

## Introduction

Proteinuria is one of the main laboratory findings in nephrology, and high urinary excretion of protein is associated with an increased risk of adverse cardiovascular and renal events in individuals with chronic kidney disease (CKD). Its identification is essential in the evaluation and treatment of CKD, as in other diseases^1,2^. However, there is no universally accepted method to assess proteinuria, and guidelines are inconsistent on whether measurement of total urine protein excretion or only urinary albumin excretion should be recommended for risk assessment and therapeutic decisions^1,2^.

Currently, there are several methods for measuring urinary protein. The most common in clinical practice are reagent strips (semi-quantitative evaluation), precipitation, and electrophoresis^3^. Tests for quantification of proteinuria can be performed in 24-hour urine or in spot samples. Although 24-hour urine tests to quantify proteinuria and albuminuria are considered more reliable, they are more prone to errors related to urine collection (pre-analytical errors). Given these limitations, the main guidelines recommend the use of isolated urine samples for routine care^2,3^. A simultaneous assessment of proteinuria and albuminuria in random urine samples through protein-to-creatinine ratio (PCR) and albumin-to-creatinine ratio (ACR), respectively, has been proposed. Both PCR and ACR in a urine sample are closely related to daily excretion of protein or albumin in grams^2–4^.

In 1983, Ginsberg et al.^4^ were the first to describe a strong correlation between PCR and 24h-urine proteinuria. Since then, this correlation has also been verified by other studies in patients with CKD (diabetic or non-diabetic), kidney transplant recipients, and pregnant women^5,6^. Similarly, studies indicate a high degree of agreement between PCR and 24-hour urine albumin excretion in different patient profiles^7,8^. In 2009, a study using receiver operator characteristic (ROC) curve analysis proved the accuracy of ACR and PCR for the assessment of albuminuria and proteinuria in outpatients^9^. Thus, there is substantial evidence to support the use of the ACR and PCR as valid surrogates for 24-hour urine measurements, and consequently the conclusions that can be drawn from them, as in the study in question.

In 1964, protein selectivity (selectivity index) was first reported to indicate the response to steroid therapy in adult nephrotic syndrome. Later, the prognostic value of selectivity was extended to predict clinical remission in other glomerular diseases, including membranous glomerulonephritis, and more recently the response to treatment and the presence of chronic lesions on renal biopsy of patients diagnosed with lupus nephritis^10^.

Urinary protein electrophoresis (UPE), another method for evaluating proteinuria (in 24-hour samples), is not only quantitative but also qualitative. It provides information about where most of the protein is coming from and its selectivity^11,12^. However, other authors have already hypothesized that the type of proteinuria, given by the electrophoretic pattern and immunofixation, can be predicted by the simple proportion of higher molecular weight proteins, such as albumin, in the total protein content in urine, i.e., the APR ratio^12^.

Thus, we pursue to validate the APR as a cheaper and readily available substitute for UPE in outpatients.

## Methods

This was a single-center, cross-sectional, retrospective, observational study. All participants were adult CKD patients admitted to the Nephrology Outpatient Clinic of a university hospital in Brazil, between January 2018 and December 2019.

### Patient Selection

All CKD patients older than 18 years were eligible to participate in this study. There was no restriction regarding gender, ethnicity, or presence of comorbidities. A cut-off limit of 18 mg of proteinuria was established, by which electrophoretic separation of protein fractions is possible. Patients below the cut-off limit were excluded. The urine samples were obtained randomly from an outpatient clinic with about 200 CKD patients during the period of UPE availability in the hospital. All patients meeting the inclusion criteria who agreed to participate in the study were allocated.

### Data Collection

The 24-hour urine samples and spot urine samples were collected from 42 eligible patients. The samples were collected at different moments, but all were collected within one month. Albumin, protein, and creatinine concentrations were measured in random urine samples, and albumin-creatinine ratio (ACR), protein-creatinine ratio (PCR), and albumin/protein ratio (APR) were calculated from those variables. In addition, urinary protein electrophoresis (UPE) was performed in 24-hour urine and used as reference.

Urine albumin concentration was determined by turbidimetric immunoassay and urine protein concentration was measured with a pyrogallol red-molybdate complex on an automatic analyzer. Urine creatinine concentration was determined by the Jaffé’s kinetic method. The ACR (mg/g) was calculated using albumin concentration (mg/dL) divided by creatinine concentration (mg/dL) and the PCR (mg/g) was calculated by protein concentration (mg/dL) divided by creatinine concentration (mg/dL). Finally, the APR is the division between ACR and PRC.

Glomerular filtration rate was estimated using the CKD-EPI equation and followed the criteria proposed by the *Kidney Disease Improving Global Outcomes* (KDIGO) for the classification of CKD^2^. Eligible patient records were reviewed for clinical and demographic data relevant to the study.

### Statistical Analysis

Categorical variables were analyzed by the softwares SPSS^®^ version 20.0 (IBM^©^, Chicago, IL, United States) and Python version 3.7 (Python Software Foundation Inc. – USA). The Shapiro-Wilk test and histogram analysis were used to test normality. The correlation between variables was obtained through Spearman’s correlation. The linear regression that generated the residuals was optimized based on the least square’s method. The non-violation of heteroscedasticity was analyzed using the Breusch-Pagan test. The nonlinear regression was optimized using the residual sum of squares. P-values < 0.05 were considered statistically significant.

### Ethical Approval

The study was approved by the Research Ethics Committee of the Universiade Federal Fluminense Medical School under the number CAE 14399513.2.0000.5243.

## Results

A total of 42 patients were analyzed, of whom half were men, most were non-white, and the median age was 56.4 years. Most of them were hypertensive (**Table 1**). The median estimated glomerular filtration rate was 24.9 mL/min/1.73 m^2^ with a standard deviation of 39.7 mL/min/1.73 m^2^ (**Table 1**). The patients were stratified in different stages of CKD during the study: 9 in stage G1, 4 in stage G2, 1 in stage G3a, 5 in stage G3b, 13 in stage G4, and 10 in stage G5 of the disease. As for APR, the median was 0.504 (interquartile range: 0.411–0.596). Finally, the albumin fraction in UPE median was 53.1% (interquartile range: 45.3–60.7).

**Table 1:** Characteristics of the Participants (N = 42)

Male, n (%)	21 (50.0)
Age (years)	56.4 (49.2–60.0)
Non-white skin color, n (%)	38 (90.5)
Hypertension, n (%)	31 (73.8)
eGFR (mL/min/1.73 m^2^)	24.9 (16.5–76.3)
Albumin-to-protein ratio	0.504 (0.411–0.596)
Albumin fraction using UPE (%)	53.1 (45.3–60.7)

Values are expressed as frequency (%) or median (interquartile range). eGFR: estimated glomerular filtration rate; UPE: urinary protein electrophoresis.

Regarding the underlying diseases of the patients in the study: 12 had diabetic or hypertensive nephropathy, 5 had rheumatological disease (lupus, mixed connective tissue disease, and Sjögren’s syndrome), 3 had renal amyloidosis, 4 had hematological disease (multiple myeloma and lymphoma), and there were also 3 cases of focal segmental glomerulosclerosis, 1 kidney disease due to non-steroidal anti-inflammatory drug abuse, 1 polycystic kidney disease, 1 CKD due to repeated urinary tract infections, and 12 remained with the diagnosis of unspecified CKD or syndromic diagnosis (nephritic or nephrotic syndrome) because they were still under diagnostic investigation.

**Figure 1:**
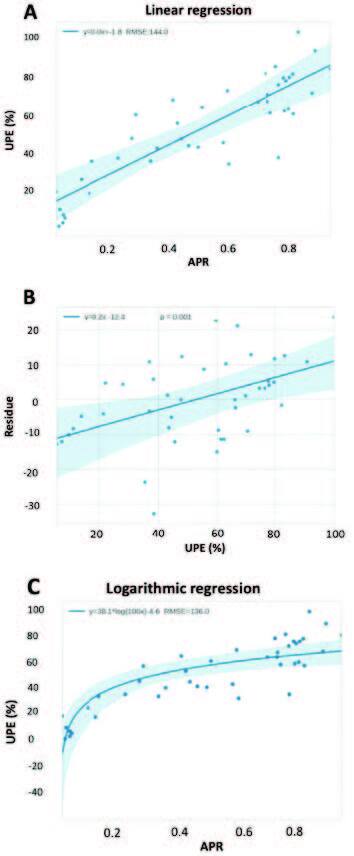
**(A)** The blue line indicates the simple linear regression obtained using the partial least squares method. The shaded region represents the 95% confidence interval of the regression. **(B)** Linear regression between the residual obtained from the regression between the APR and the UPE and with the UPE itself. This graph shows a certain degree of dependence between the error and the percentage obtained from electrophoresis. **(C)** Logarithmic regression between albumin fraction using urinary protein electrophoresis and the APR. UPE = Urinary protein electrophoresis; APR = albumin/protein ratio or index.

The linear regression model with the ordinary least square’s method with UPE as dependent variable and APR as independent variable demonstrated an angular coefficient of 72.1 and constant of −4.6, both significant in the two-tailed t-test <0.001, bootstrap sample. The regression model was represented by the following equation: y = 72.1x − 4.6. The Breusch-Pagan test for heteroscedasticity had a p-value of 0.397, and the null hypothesis of homoscedasticity could not be rejected; furthermore, the residuals showed normality and independence according to the Durbin-Watson test. Despite the non-rejection of the homoscedasticity of the residuals, a dependence of this error on UPE was observed, since the linear regression line had an angular coefficient of 0.2 and a constant value of −12.4, with a p-value of 0.001 in the two-tailed t-test (**Figure 1**).

The logarithmic regression model optimized by the residual sum of squares had a beta of 38.1 and an alpha value of −4.6, both significant in the two-tailed t-test (P < 0.001), bootstrap 1000 samples. The model was represented by the following equation: y = 38.1*log(100x) − 46. It showed a lower value of the square root of the mean error (SRME), 136.0, compared to that of the linear regression with RMSE of 144, showing a better fit to the data, but this trend cannot be generalized due to the little statistical power in detecting small differences. (**Figure 1**).

Regarding the inferential analyses, a strong Spearman’s correlation of non-linear order was observed between ARP and albumin fraction using EPU; r = 0.84 (95% confidence interval 0.75–0.92), p = 0.001. Both logarithmic and linear regression showed good fit to the data, but logarithmic regression showed better visual support and higher correlation in previous attempts compared to linear regression (**Figure 1C**).

## Discussion

The present study showed that APR values had a strong statistical correlation with albumin fraction in the UPE. Thus, a higher albumin content as a proportion of total urine protein content could reflect a predominantly glomerular pattern, whereas a lower albumin content could reflect a tubulointerstitial pattern of urine protein loss. Studies in pediatric populations have found a significantly lower APR index associated with tubular and non-primary glomerular disease^13,14^. These were the first studies to point to the use of APR in determining the type of proteinuria, thus also inspiring us to use it a substitute for the UPE.

In 2012, Smith et al.^12^ examined the relationship between ACR, PCR, and APR with UPE patterns in a cohort of urine samples. In the ROC curve analysis, the area under the curve of APR was 0.84 for predicting the pattern of tubular proteinuria in UPE. The APR had an equal prediction to the UPE, a tubular pattern of protein in the urine. In this validation cohort, an APR cut-off point of <0.40 had an 88% sensitivity and 99% specificity for the diagnosis of primary tubulointerstitial disorders on renal biopsy^12^.

However, a 2016 Korean study involving patients diagnosed with multi-stage chronic kidney disease obtained opposite results. Hong et al.^15^ compared the diagnostic usefulness of the APR index compared with the UPE. The correlation between these variables was assessed, but the result was not very significant, probably due to the profile of the patients, with a correlation r^2^ = 0.33 and p-value < 0.0001.

## Conclusion

There is a relationship of logarithmic order between the APR and UPE, and more importantly, a very strong correlation also exists between the albumin fraction observed in the UPE and the APR ratio in outpatients. Therefore, the type of proteinuria (selectivity) can be inferred by means of this APR, which is a cheaper and readily available compared with UPE. However, more robust analyses are needed to validate the use of APR as an alternative to UPE and its use in other populations.

## References

[1] Weaver R, James M, Ravani P, Weaver CGW, Lamb EJ, Tonelli M (2020). Estimating urine albumin-to-creatinine ratio from
protein-to-creatinine ratio: development of equations using same-day
measurements.. J Am Soc Nephrol..

[2] Kidney Disease Improving Global Outcomes. (2013). Clinical practice guideline for the evaluation and management of
chronic kidney disease.. Kidney Int Suppl..

[3] Viswanathan G, Upadhyay A. (2011). Assessment of proteinuria.. Adv Chronic Kidney Dis..

[4] Ginsberg J, Chang B, Matarese R, Garella S. (1983). Use of single voided urine samples to estimate quantitative
proteinuria.. N Engl J Med..

[5] Ruggenenti P, Gaspari F, Perna A, Remuzzi G. (1998). Cross sectional longitudinal study of spot morning urine protein:
creatinine ratio, 24-hour urine protein excretion rate, glomerular
filtration rate, and end stage renal failure in chronic renal disease in
patients without diabetes.. BMJ.

[6] Torng S, Rigatto C, Rush DN, Nickerson P, Jeffery JR. (2001). The urine protein to creatinine ratio (P/C) as a predictor of
24-hour urine protein excretion in renal transplant
patients.. Transplantation.

[7] Neithardt AB, Dooley SL, Borensztajn J. (2002). Prediction of 24-hour protein excretion in pregnancy with a
single voided urine protein-to-creatinine ratio.. Am J Obstet Gynecol..

[8] Zelmanovitz T, Gross J, Oliveira J, Paggi A, Tatsch M, Azevedo MJ (1997). The receiver operating characteristics curve in the evaluation of
a random urine specimen as a screening test for diabetic
nephropathy.. Diabetes Care.

[9] Guy M, Borzomato  J, Newall R, Kalra PA, Price CP (2009). Protein and albumin-to-creatinine ratios in random urines
accurately predict 24 h protein and albumin loss in patients with kidney
disease.. Ann Clin Biochem..

[10] Hasegawa T, Suzuki K, Kaneko Y, Takeuchi T (2017). Proteinuria selectivity index as a prognostic biomarker in lupus
nephritis.. Lupus..

[11] Brocklebank T, Cooper EH, Richmond K (1991). Sodium dodecyl sulphate polyacrylamide gel electrophoresis
patterns of proteinuria in various renal diseases of
childhood.. Pediatr Nephrol..

[12] Smith ER, Cai MM, McMahon LP, Wright DA, Holt SG (2012). The value of simultaneous measurements of urinary albumin and
total protein in proteinuric patients.. Nephrol Dial Transplant..

[13] Abitbol CL, Chandar J, Onder AM, Nwobi O, Montane B, Zilleruelo G (2006). Profiling proteinuria in pediatric patients.. Pediatr Nephrol..

[14] Lun A, Suslovych M, Drube J, Ziebig R, Pavicic L, Ehrich JH (2008). Reliability of different expert systems for profiling proteinuria
in children with kidney diseases.. Pediatr Nephrol..

[15] Hong D, Oh I, Park JS, Lee CH, Kang CM, Kim GH (2016). Evaluation of urinary indices for albuminuria and proteinuria in
patients with chronic kidney disease.. Kidney Blood Press Res..

